# *In Vitro* Pollen Viability and Pollen Germination in Cherry Laurel (*Prunus laurocerasus* L.)

**DOI:** 10.1155/2014/657123

**Published:** 2014-10-22

**Authors:** Melekber Sulusoglu, Aysun Cavusoglu

**Affiliations:** ^1^Arslanbey Agricultural Vocational School, Kocaeli University, 41285 Kocaeli, Turkey; ^2^Department of Horticulture, Graduate School of Natural and Applied Sciences, Kocaeli University, 41380 Kocaeli, Turkey

## Abstract

Pollen quality is important for growers and breeders. This study was carried out to determine *in vitro* pollen viability and pollen germination in seven genotypes of cherry laurel (*Prunus laurocerasus* L.). Two pollen viability tests, TTC (2,3,5-triphenyl tetrazolium chloride) and IKI (iodine potassium iodide), were used. Pollen traits of genotypes were studied using an *in vitro* medium containing 0%, 5%, 10%, 15%, and 20% sucrose to determine the best sucrose concentrations for germination. In the second step, the germinated pollen was counted 1, 4, 6, 10, 12, 24, and 48 hours later until there was no further germination. The viability rates were different according to genotypes and tests used. The IKI and TTC staining tests and pollen germination had low correlation (*r* ^2^ = 0.0614 and *r* ^2^ = 0.0015, resp.). Painted pollen rate was higher and pollen was well-stained with IKI test and pollen viability estimated with TTC staining test was better than that estimated with the IKI staining test. 15% sucrose gave the best germination rates in most of the genotypes. Pollen germination rates were recorded periodically from one hour to 48 hours in 15% sucrose and the results showed that pollen germination rates increased after 6 hours of being placed in culture media.

## 1. Introduction

The development of reliable methods for determining the functional quality of pollen helps in monitoring pollen vigor during storage, genetics and pollen-stigma interaction studies, crop improvement and breeding, and incompatibility and fertility studies [1–4]. The quality of pollen is assessed on the basis of viability and vigor of the pollen grain. Pollen vigor refers to the speed of germination of pollen grains and the rate of pollen tube growth [5].* In vitro* pollen germination tests have been used to determine the germination percentage of pollen and can also be used for assessing pollen vigor by monitoring the rate of germination over a period of time or the length of pollen tubes [6]. Pollen which could not germinate usually shows poor tube growth which is likely to be ineffective in causing fertilization. In the breeding of fruit species it is very important to use a suitable method for determining pollen viability [7].* In vitro* germination tests have been used to indicate viability of pollen [8]. In general, there is a linear relationship between pollen viability and germination capability in many fruit species [1]. However in some cases, different results may be obtained in cultivars with staining tests. Therefore, to determine the actual amount of viable pollen, germination tests are necessary.

The material in this study is cherry laurel (*Prunus laurocerasus* L.), belonging to* Rosaceae* family. It is one of the fruit species native to southeastern Europe and Asia Minor around the Black Sea in the Caucasus and Transcaucasia mountains of Anatolia, Bulgaria, and Serbia [9].* Prunus laurocerasus* is an evergreen plant with small cherry fruits, a few centimeters in diameter. The fruit color is red to black when ripe. Before maturity fruits are astringent but become sweet and reasonably pleasant when fully ripe. The stones when ground into powder are very good for bronchitis [10]. Cherry laurel fruits are considered a significant source of phenolic compounds and anthocyanins [11, 12], and it is one of the most widely used ornamental plants for landscaping [13].

It is widely spread in the northern part of Turkey and there are many cultivars which show different traits. As an alternative fruit, with a different taste, it has commercial value in Turkey and many gardens are planted in selected 4 cherry laurel genotypes. Some breeding and cultivation for ornamental studies have just started in United States and Europe [14–16]. Cherry laurel yield amount varies from year to year; that is, the trees tend to have high production in one year followed by low production the following year. The most important factors affecting fruit set are late spring frost, rainy spring weather during bloom, heat stress, and drought [10]. Breeding to obtain cultivars with high fruit quality has rarely been attempted [12, 17–19]. Cherry laurel is a self-incompatible species and needs cross-pollination for fruit set according to our observations. There is usually severe fruit drop leaving no fruit on a shoot. One of the important factors for fertilization success is pollen viability and germination; therefore pollen performance may have a significant role in pollination for adequate fruit yield [20, 21]. Information on pollen biology of cherry laurel is very limited and for this purpose, the aim of the study was to determine the pollen viability and* in vitro* germination rate of cherry laurel pollen.

## 2. Materials and Method

The study was conducted over 2011-2012 to evaluate the pollen quality of seven cherry laurel genotypes. These seven genotypes were selected from the selection study where 40 cherry laurel genotypes were evaluated between 2008 and 2010 [22]. Unopened flowers were collected at balloon stage in April from all sides of one tree (each genotype/one tree). Petals and sepals were separated and anthers were isolated from flower buds and placed on a black paper under an incandescent lamp on a table overnight. In the next day, the pollen grains were collected in a small glass vial.

Pollen viability was estimated using two staining techniques, IKI (iodine potassium iodide) [23] and TTC (2,3,5-triphenyl tetrazolium chloride) [24–26]. 1% TTC (0.2 g. TTC and 12 g sucrose dissolved in 20 mL distilled water) was used in the first step and a drop of the mixture was dropped on a microscope slide and the pollen spread with a slim brush and covered with a coverslip. Pollen viability counts were made after two hours; then pollen was placed on a TTC solution. Pollen grains stained that orange or bright red color were counted as viable. In the second staining method, 1 g potassium iodide and 0.5 g iodine were dissolved in 100 mL distilled water for the IKI solution. Pollen viability counts were made five minutes after pollen was placed on an IKI solution. Pollen grains stained dark (dark red or brown color) were counted as alive.

Stain tests are faster and easier than pollen germination tests but in some cases germination tests are necessary to observe the actual viability of pollen. Studies on pollen viability in* Prunus* that* in vitro* germination by an agarose-sucrose medium gave good results [27] and we used the agarose-sucrose medium method for the pollen germination treatments. In the first step of pollen germination studies, different sucrose concentrations (0, 5, 10, 15, and 20%) were added into media together with 0.5% agar and 5 ppm boric acid (H_3_BO_3_) to determine the effects of sucrose concentrations. Pollen germination was observed at the end of 24-hour incubation period. In the next study pollen germination rate was determined during the 48-hour incubation period, in the best level of sucrose (15%), in an oven, at 22°C in the dark. The germinated pollen was counted 1, 4, 6, 10, 12, 24, and 48 hours after incubation.

In both germination treatments, the pollen grains were considered germinated when the pollen tube length reached pollen diameter [28]. The experiments were designed as completely randomized blocks with three replications. Randomly selected visual areas, including about 100 pollen grains, were counted in each replicate. All observations of viability and germination were made at ×100 magnification using a light microscope.

In both years (2011 and 2012), pollen viability and germination rates were very close to each other. All the data collected were combined and the means were used in the statistical analysis. Statistical analysis was performed in the Minitab 17 Statistical program. One-way ANOVA was used for the analysis of viability and germination rates and different means were compared with Duncan's Multiple Range Test at *P* < 0.05. The values for viability and pollen germination were transformed to arcsin square root values before statistical analysis.

Correlation analysis was performed to determine the relationship between pollen viability and pollen germination (after 48 hours). These correlations were evaluated using MINITAB 17 statistical program (*P* ≤ 0.05) for TTC and IKI staining tests.

## 3. Results and Discussion

**Figure 1:**
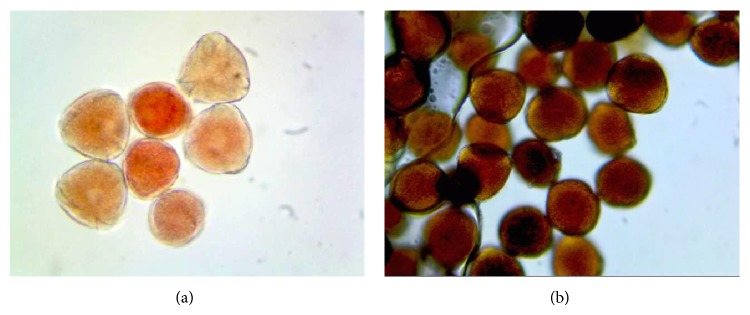
TTC (a) and IKI (b) painted pollen of cherry laurel (×400).

The results of TTC and IKI staining tests are shown in Table 1. The interaction of genotypes × staining tests was statistically significant and there were slight differences in the pollen viability percentages of genotypes in both staining tests. IKI gave the highest pollen viability rates and the genotypes stained better with IKI solution than pollen stained with 1% TTC (Figures 1(a) and 1(b)).

The cherry laurel genotypes exhibited differences in pollen viability depending on the staining method. In the TTC test, genotype 36 had the highest pollen viability (97.69%) but this result was not different statistically from other genotypes. According to viability rates obtained with IKI, the lowest percentage of viable pollen was found to be 81.25% in genotype 4 and this result is statistically significant (Table 1). Similar differences according to genotypes and staining tests have been observed in other studies [27, 29]. Determining the viability of pollen grains is an easy method to increase the efficiency of breeding programs and for selection of suitable pollinizers while orchards are being established [30, 31]. In this study, pollen viability of the genotypes was very high in both staining tests; therefore, all genotypes can be considered as good pollinators [32].

Generally pollen grains require water for the formation of a pollen tube and excellent germination was obtained in sugar media with or without a lower percentage of agar [33]. Depending on the sucrose level, cherry laurel pollen exhibited different germination rates. The interaction between sucrose concentration and genotypes of cherry laurel was not statistically significant, while the effect of sucrose concentration was found statistically significant at *P* < 0.05 level. Pollen germination rates increased up to 15% sucrose concentration and decreased at 20% sucrose concentration except in genotypes 4 and 34 (Table 2).

**Figure 2:**
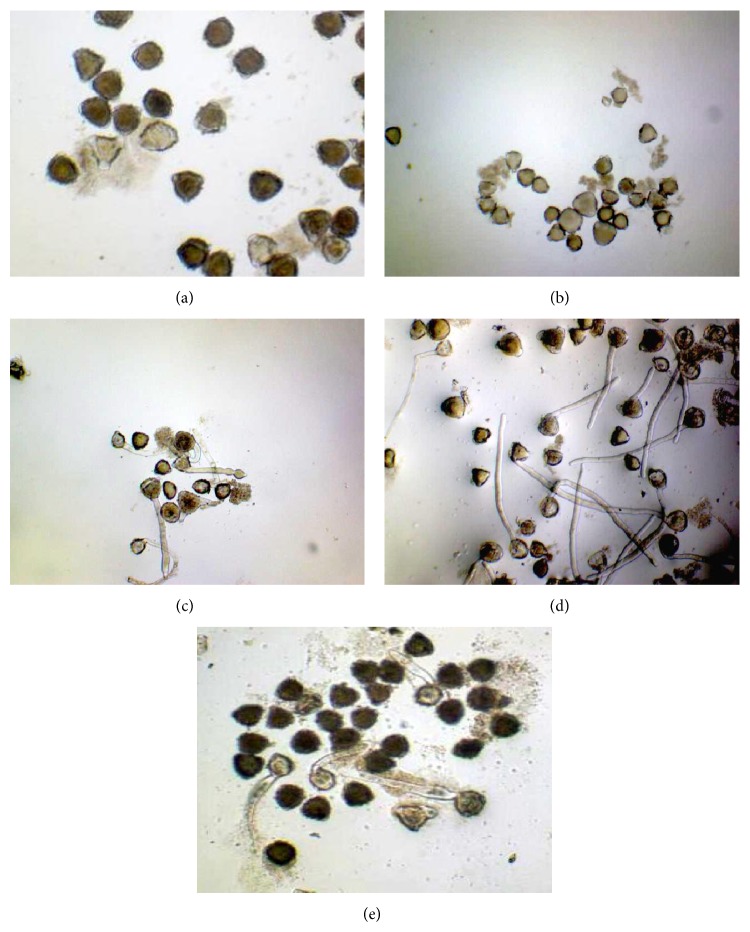
Pollen germination in media containing different sucrose concentrations after 24 hours of germination ((a) 0%, (b) 5%; (c) 10%; (d) 15%, and (e) 20% sucrose) (×100).

**Figure 3:**
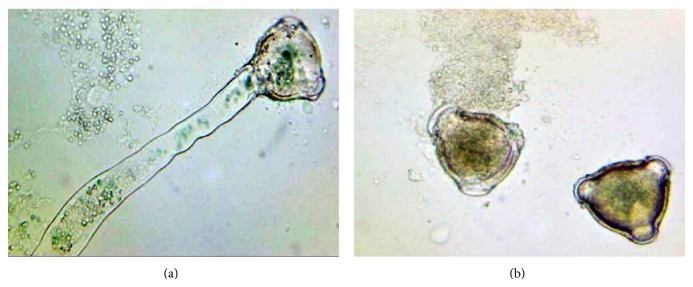
Pollen tube growth on germinated pollen (a); ungerminated pollen without a pollen tube (b) (×400).

Pollen grains did not develop a pollen tube in 0% and 5% sucrose including media, or there was only a very small tube and tube elongation increased with increasing amount of sucrose in the culture media (Figures 2(a), 2(b), 2(c), and 2(d)). Pollen tube formation was inhibited again in 20 g/L sucrose including media, and pollen tube length was decreased too (Figure 2(e)). In Figure 3, pollen tube formation of cherry laurel was given (Figure 3(a)) and ungerminated pollen without a pollen tube (Figure 3(b)) in the culture media was shown.

With genotype 34, 10% sucrose concentration gave the highest germination percentage and increasing concentration of sucrose affected negatively the germination rate of the pollen. Pollen germination rates were increased with the increasing concentrations of sucrose in genotype 4. Differences among sucrose means were found statistically significant and the lowest germination percentages were observed in sucrose free media in all genotypes. Previous studies have shown that the most suitable concentration of sucrose for stone fruit pollen germination under* in vitro* conditions was 15% or 20% sucrose [27, 34–37]. These results are in agreement with our findings.

According to personal observations, pollen grain structure exhibited high morphological homogeneity and it could be used as a good property for fertilization biology [38].

**Figure 4:**
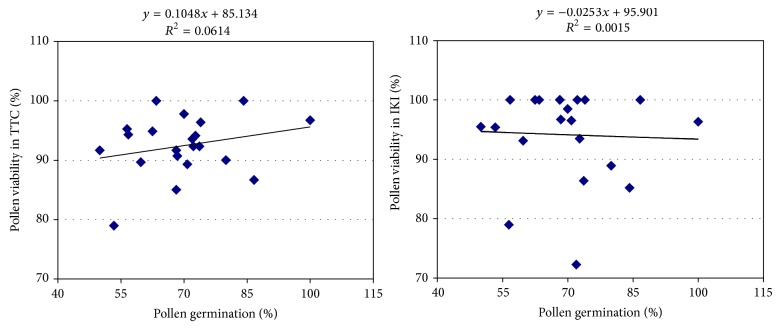
Correlation between the pollen staining test and pollen germination rates.

The correlations between pollen viability and germination tests were also determined and were not significant in TTC and IKI tests (*r* ^2^ = 0.0614 and *r* ^2^ = 0.0015, resp.) (Figure 4). Previous studies to determine the relationship between pollen viability and germination tests in fruit species showed different results. For example, viability as determined by TTC staining tests in apple, pear, peach, and grape pollen did not show good correlation with the actual germination rate [25]. However, Werner and Chang [34] observed that the correlation between the MTT (Baker's and X-Gall) stain test and pollen germination in peach was significant but the IKI test was not. Parfitt and Ganeshan [27] have established that the pollen stain tests (including TTC) are not reliable or consistent and are not positively correlated with* in vitro* germination tests. These results are in agreement here with our findings. These stains may therefore be used to determine pollen viability in these species to provide only a rough estimate of viability.

Incubation time of pollen is important for* in vitro* pollen germination and in the second step of the germination studies, incubation time effects were significant (Table 3). Pollen tubes began to grow approximately 30 minutes after being placed in the growing medium and reached maximum germination at the end of 48 hours (Table 3). As reported in another study, the highest germination was obtained after 48 hours of incubation period for cherry cultivars [29]. Differences between 24-hour and 48-hour pollen germination rates were statistically not significant but pollen tube lengths dramatically increased over time (data not presented). 24 hours may therefore be enough for germination of cherry laurel pollen.

**Figure 5:**
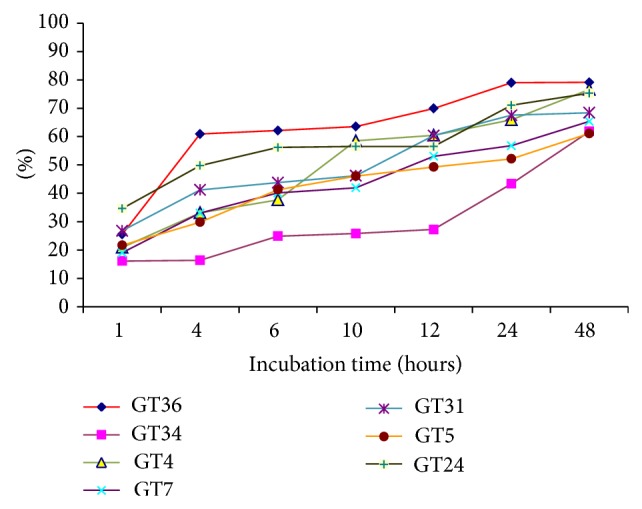
Germination percentage of cherry laurel genotypes over the incubation period.

In the 48-hour germination period, pollen germination rates were different among the genotypes and this was statistically significant at *P* < 0.05 (Table 3). Genotype 36 germinated faster than other genotypes and gave the highest germination rate (79.14%) at the end of 48 hours. According to mean germination values, this genotype showed statistically higher germination rates than other genotypes over the counting period (Figure 5). There are reports in the literature on the occurrence of variation in* in vitro* pollen germination of fruit cultivars, such as sweet cherry, caprifig, and medlar [29, 39, 40], and the results presented here corroborate those studies.

## 4. Conclusions

In this study, results showed pollen viability and germination rates in various conditions for genotypes of cherry laurel. Results of this study indicated that TTC and IKI staining tests could be used to determine the viability of cherry laurel pollen while TTC gave results close to real germination rates. The results of viability tests were close among the cherry laurel genotypes and viability rates were very high. 15% sucrose was the best sucrose concentration for germination and the appropriate germination time was 24 hours by both observation and counting. The results presented here are the first observation on the cherry laurel pollen viability and germination rate that will help in cherry laurel reproduction and artificial pollination studies.

## Figures and Tables

**Table 1:** Pollen viability of cherry laurel genotypes determined by TTC and IKI tests.

Genotypes∗	TTC (%)	IKI (%)	Mean of genotypes (%)
GT4	95.29^abA^	81.25^bB^	88.27
GT5	86.87^abA^	93.24^aA^	90.06
GT7	88.78^abB^	97.70^aA^	93.24
GT24	88.89^abB^	97.73^aA^	93.31
GT31	95.59^abA^	97.30^aA^	96.45
GT34	93.95^abA^	92.98^aA^	93.47
GT36	97.69^aA^	98.77^aA^	98.23

Mean	92.44	94.14	General mean: 93.29

^*^Genotypes names were given as in the selection study [22].

Values within the same column (genotypes) with different lower-case letters and values within the same row (staining tests) with different capital letters are significantly different (*P* < 0.05).

**Table 2:** Effect of sucrose concentrations on agar plate media on pollen germination of cherry laurel.

Genotypes	Sucrose (%)					Mean of genotypes
	0	5	10	15	20	
GT4	26.84	38.33	56.31	64.98	69.36	51.16
GT5	18.68	37.71	41.78	69.66	60.47	45.66
GT7	15.86	32.84	50.80	67.84	59.92	45.45
GT24	44.30	52.97	56.40	71.03	53.81	55.70
GT31	23.32	33.84	47.37	69.51	47.32	44.27
GT34	32.56	34.98	65.46	60.75	55.71	49.89
GT36	39.90	47.04	55.75	72.13	68.26	56.62

Mean	28.78^c^	39.67^bc^	53.41^ab^	67.99^a^	59.26^a^	General mean: 49.82

Values with different lower-case letters are significantly different at *P* < 0.05.

**Table 3:** Germination rate of cherry laurel genotypes within 48 hours.

Genotypes	Germination time (hours)							Mean of genotypes
	1	4	6	10	12	24	48	
GT4	20.92	33.11	37.71	58.49	60.45	65.93	76.63	50.46ab
GT5	21.66	29.83	41.27	46.06	49.28	52.15	61.11	43.05bc
GT7	19.01	33.00	40.12	41.94	53.06	56.78	65.37	44.18bc
GT24	34.61	49.77	56.16	56.53	56.53	71.04	75.31	57.14ab
GT31	26.78	41.19	43.78	46.21	60.37	67.55	68.41	50.61ab
GT34	16.11	16.40	24.89	25.8	27.26	43.38	61.82	30.81c
GT36	25.50	60.91	62.13	63.56	69.91	79.01	79.14	62.88a

Mean	23.51^d^	37.74^cd^	43.72^c^	48.37^bc^	53.84^bc^	62.26^ab^	69.68^a^	48.45

Values with different lower-case letters are significantly different at *P* < 0.05.
